# WNT/β-Catenin signaling pathway regulates non-tumorigenesis of human embryonic stem cells co-cultured with human umbilical cord mesenchymal stem cells

**DOI:** 10.1038/srep41913

**Published:** 2017-02-03

**Authors:** Yu-Hsun Chang, Tang-Yuan Chu, Dah-Ching Ding

**Affiliations:** 1 Department of Pediatrics, Buddhist Tzu Chi General Hospital, Hualien, Taiwan, R.O.C; 2 Lab of stem cell research, Department of Research, Buddhist Tzu Chi General Hospital, Hualien, Taiwan, R.O.C; 3 Institue of Medical Science, Tzu Chi University, Hualien, Taiwan; 4 Department of Obstetrics and Gynecology, Buddhist Tzu Chi General Hospital, Taiwan, R.O.C; 5 Cervical Cancer Prevention Center, Department of Research, Buddhist Tzu Chi General Hospital, Hualien, Taiwan, R.O.C

## Abstract

Human pluripotent stem cells harbor hope in regenerative medicine, but have limited application in treating clinical diseases due to teratoma formation. Our previous study has indicated that human umbilical cord mesenchymal stem cells (HUCMSC) can be adopted as non-teratogenenic feeders for human embryonic stem cells (hESC). This work describes the mechanism of non-tumorigenesis of that feeder system. In contrast with the mouse embryonic fibroblast (MEF) feeder, HUCMSC down-regulates the WNT/β-catenin/*c*-myc signaling in hESC. Thus, adding β-catenin antagonist (FH535 or DKK1) down-regulates β-catenin and *c*-myc expressions, and suppresses tumorigenesis (3/14 vs. 4/4, *p* = 0.01) in hESC fed with MEF, while adding the β-catenin enhancer (LiCl or 6-bromoindirubin-3′-oxime) up-regulates the expressions, and has a trend (*p* = 0.056) to promote tumorigenesis (2/7 vs. 0/21) in hESC fed with HUCMSC. Furthermore, FH535 supplement does not alter the pluripotency of hESC when fed with MEF, as indicated by the differentiation capabilities of the three germ layers. Taken together, this investigation concludes that WNT/β-catenin/*c*-myc pathway causes the tumorigenesis of hESC on MEF feeder, and β-catenin antagonist may be adopted as a tumor suppressor.

Stem cell research has been widely performed in recent decades, and holds much hope for regenerative medicine. The most promising stem cells are pluripotent human embryonic stem cells (hESC) and human-induced pluripotent stem cells (iPSC)^1^. However, the risk of teratoma formation has largely restricted the clinical application of stem cells in regenerative medicine^2^. The mechanism of tumorigenesis in these pluripotent hESs needs to be understood in order to identify a method of propagating ES cells that minimizes the risk of teratoma formation while maintaining their pluripotency.

Human umbilical cord mesenchymal stem cells (HUCMSC) from epiblasts of the human embryo can be robustly isolated from the Wharton’s jelly of the umbilical cord after birth^3^,^4^,^5^,^6^. Previous studies have adopted HUCMSC as a feeder to support the growth of hESC^3^,^7^. Our previous research found that co-culturing of mitomycin-inactivated HUCMSC with hESC maintained the pluripotency features of hESC after long-term propagation. Notably, this HUCMSC-cocultured hESC does not form teratoma in xenograft, but the teratogenic phenotype can be restored upon transient co-culturing with MEF. Thus, HUCMSC transmits a tumor suppressive signal to the co-cultured hESC. Interestingly, among the four pluripotency-conferring genes, the *CMYC* oncogene is downregulated in hESC with HUCMSC co-culture. This study assumes that HUCMSC may confer a signal that down-regulates *CMYC* in hESC.

Myc is a downstream target of β-catenin^8^, which is one of the major pathways fundamental for maintaining pluripotency and tumorignesis in hESC^9^,^10^,^11^,^12^. Cytosolic β-catenin is constitutively phosphorylated at specific serine residues by an enzymatic complex of adenomatous *polyposis coli* (APC), Axin and the kinases glycogen synthase kinase-3β (GSK-3β) and casein kinase I, marking it for ubiquitin-mediated proteolysis. Binding of Wnt to the cell surface Frizzled receptors and LRP5/6 co-receptors protects β-catenin from degradation, and acts on its targets, including *c*-myc, to promote cell cycle progression and inhibit apoptosis^13^,^14^,^15^. DKK1 impedes the signaling pathway by isolating the LRP6 co-receptor^16^. FH535 is a small molecule that inhibits the Wnt/β-catenin signaling pathway by antagonizing β-catenin/Tcf/LEF (T-cell factor/lymphoid enhancer factor)-mediated transcription^17^, thereby inhibiting tumor cell proliferation^17^.

This investigation explores the signaling pathway responsible for the HUCMSC-mediated down-regulation of *c*-Myc and the non-tumorigenic feature of hESC. We found that β-catenin signaling is the main factor controlling tumorigenesis, and that its inhibition mimics the tumor suppressor activity of HUCMSC.

## Results

### HUCMSC feeder inhibits tumorigenesis of hESC via the β-catenin/c-myc signaling pathway

**Figure 1:**
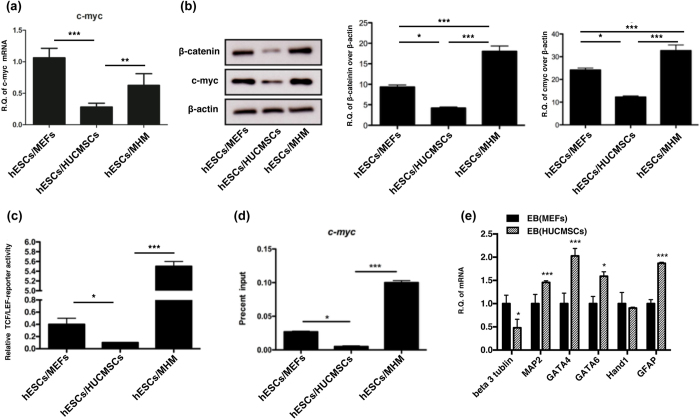
Down-regulation of β-catenin and *CMYC* in hESC co-cultured with HUCMSC. (**a**) qRT-PCR of *c-myc* of hESC/MEF, hESC/HUCMSC and hESC/MHM. (**b**) Western blotting analysis of β-catenin and *c*-myc of hESC/MEF, hESC/HUCMSC and hESC/MHM. Further shifting to MEF co-culture (hESC/MHM) reversed these expressional changes (**a**,**b**). (**c**) TCF/LEF activity of hESC cultured on different condition was measured by luciferase assay, and Firefly luciferase activity was normalized to Renilla luciferase activity, which was adopted as internal control. Values are shown as the mean of three replicates ± standard deviations. (**d**) Real-time PCR analysis of DNA fragments precipitated in a ChIP assay by using a β-catenin antibody. Primers designed for the 5′ promoter of *c-myc* were adopted to detect specific β-catenin binding. Data are represented as percentage input. Error bars represent SEM. (**e**) Three germ-layer differentiation gene expressions of embryoid body (EB) derived from hESC cultured on MEF and HUCMSC were compared by qRT-PCR (ectoderm: *β-3-tubulin, MAP2, GFAP*; endoderm: *GATA4*; mesoderm: *GATA6, Hand1*). *p < 0.05, **p < 0.01, ***p < 0.001. All cropped blots were run under the same experimental conditions in (**b**).

To identify the non-tumorigenetic signaling of HUCMSC-feeder to hESC, the expressions of β-catenin and *c*-myc in the three types of co-culture feeders were first investigated. Shifting from the MEF feeder to the HUCMSC feeder reduced the expression of mRNA and protein of β-catenin in hESC. The expression of β-catenin rebounded after shifting back to the MEF feeder (Fig. 1b). Similarly, changes to the *c*-myc expression in hESC depended on the feeder, with down-regulation occurring when using the HUCMSC feeder, and up-regulation upon shifting back to MEF (Fig. 1a,b).

A reporter assay of the key β-catenin target gene TCF/LEF was performed to investigate the downstream target of β-catenin transactivation. The hESC cultured on HUCMSC had a significant lower TCF/LEF transactivating activity than the MEF-feeder. The activity rebounded significantly upon turning back to the MEF feeder (Fig. 1c). A chromatin immunoprecipitation (ChIP) assay further confirmed the binding of β-catenin to the promoter sequences of *CMYC* in the hESC/MEF culture, and the same reduction in the hESC/HUCMSC culture and rebounding when turning back to MEF feeder (Fig. 1d). The differentiation status of hESC in MEF or HUCMSC feeder in the embryoid body (EB) state was also tested. Experimental results show expressions of genes of the three germ layers, including *beta-3-tubulin, MAP2, GFAP* (ectoderm); *GATA4* (endoderm); *GATA6, Hand1* (mesoderm) were not altered in hESC cultured on either MEf or HUCMSCs feeder (Fig. 1e). Furthermore, the EB of hESC cultured on HUCMSC had even higher expressions of *MAP2, GATA4, GATA6* and *GFAP* than that cultured on MEF.

### Lithium Chloride (LiCl) and BIO (6-bromoindirubin-3′-oxime) up-regulated the c-myc in hESC/HUCMSC *in vitro* and *in vivo*

**Figure 2:**
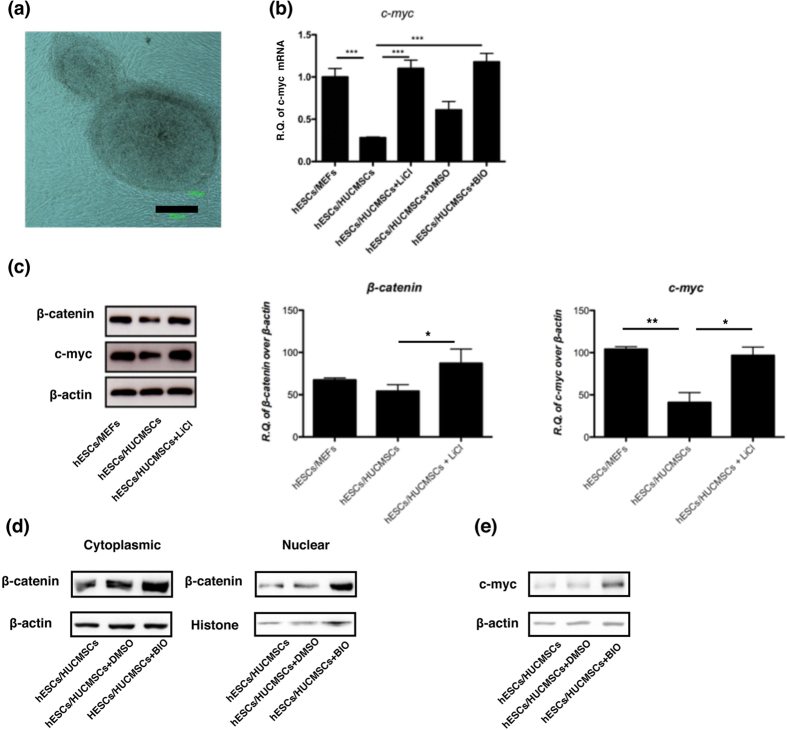
The β-catenin signaling activator LiCl and BIO up-regulates β-catenin and *CMYC* in hESC/HUCMSC. After 10 mM LiCl treatment for 24 hours, hESC/HUCMSC maintained a normal morphology for embryonic stem cells (**a**). Expressions of *CMYC* after either LiCl (10 mM) or BIO (5 μM) treatment were up-regulated at both mRNA (**b**) and protein (**c**,**e**) levels. (**d**) Western blotting analysis of nuclear translocation of active β-catenin in response to BIO 5 μM treatment for 24 hours. Scale bar = 100 μm. *p < 0.05, **p < 0.01, ***p < 0.001. All cropped blots were run under the same experimental conditions in (**c**–**e**).

To determine whether *c*-myc is up-regulated via the canonical β-catenin signaling pathway in hESC/HUCMSC, LiCl and BIO were applied to increase the cytoplasmic β-catenin. Treatment with 10 mM LiCl treatment did not change the cell morphology of hESC/HUCMSC (Fig. 2a), but significantly increased the protein level of β-catenin, and the mRNA and protein levels of *CMYC* (Fig. 2b,c). Treatment with BIO also showed the same results (Fig. 2d,e).

**Figure 3:**
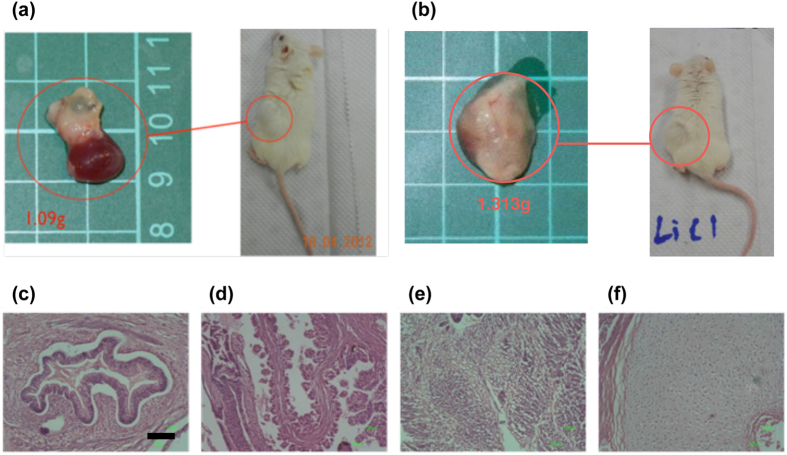
LiCl-treatment promotes teratoma formation in hESC/HUCMSC xenograft. Tumor formation was observed 12 wks after sc injection of 1 × 10^5^ hESC cells on HUCMSC feeder into NOD/SCID mice. Two of 7 mice grew tumors when injected with LiCl-treated hESC/HUCMSC (**a**,**b**). Hematoxylin and eosin stain of the re-sected tumor indicated a histology mature teratoma with evident endoderm (**c**,**d**), ectoderm (**e**) and mesoderm (**f**) components.

The regain of tumorigeneity in the xenograft of hESC/HUCMSC after activation of β-catenin by LiCl was also studied. After 10-hour treatment with LiCl (10 mM) in hESC/HUCMSC, teratoma formed in 2/7 (28.6%) grafted mice, compared to 0/21 (0%) in the non-treated group (Fig. 3A and Table 1) (p = 0.056). The grown teratomas indicate typical tissues of the three germ layers (Fig. 3c–f).

### FH535 inhibited and LiCl promoted teratoma formation through the β-catenin/c-myc signaling pathway *in vivo*

**Figure 4:**
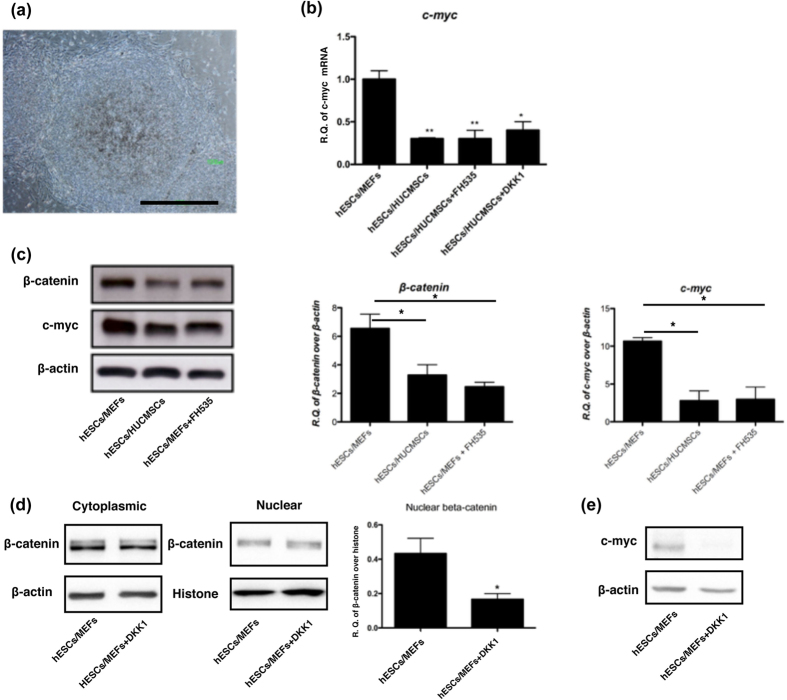
β-catenin transactivation antagonist FH535 and DKK1 inhibits β-catenin and *CMYC* expression in hESC co-cultured with MEF. (**a**) The typical morphology of hESC remained unchanged after treating hESC/MEF with 10 μM FH535 for 8 hours. Scale bar = 1000 μm. (**b**) qRT-PCR analysis *CMYC* mRNA expression of hES/MEF treated by FH535 (10 μM) and DKK1 (250 ng/ml) for 24 hours. (**c**) Western blotting analysis of Myc protein was down-regulated to a level equivalent to that in hES/HUCMSC. Quantitative expression of β-catenin and *c*-myc protein in three independent experiments is shown in the two right-hand panels. (**d**) Western blotting analysis of nuclear translocation of active β-catenin in response to DKK1 (250 ng/ml) treatment of hES/MEF for 24 hours. Quantification of nuclear fraction of β-catenin (in triplicate) is shown in the right panel. (**e**) Western blotting analysis of *c*-myc in hESC/MEF after treating DKK1 for 24 hours. *p < 0.05, **p < 0.01, ***p < 0.001. All cropped blots were run under the same experimental conditions.

To further confirm the tumorigenic role of β-catenin/*c*-myc signaling, the hESC/MEF were treated with the β-catenin signaling inhibitor FH535, and subjected to xenograft in NOD/SCID mice. After 3 months, FH535 significantly inhibited teratoma formation of hESC/MEF. Only 3 of 14 (21.4%) injection sites developed teratoma, compared to 4 of 4 (100%) in the non-treated group (*p* = 0.011, Table 2). The β-catenin mediated myc effect was also observed in the hESC culture. As demonstrated in Fig. 4, 24 hours treatment with FH535 or DKK1 led to down-regulation of the downstream β-catenin and *c*-myc protein was evident in hESC/MEF (Fig. 4), but did not change the morphology of hES cells (Fig. 4a).

### Adding FH535 to hESC/MEF did not alter the pluripotency or differentiation capability

**Figure 5:**
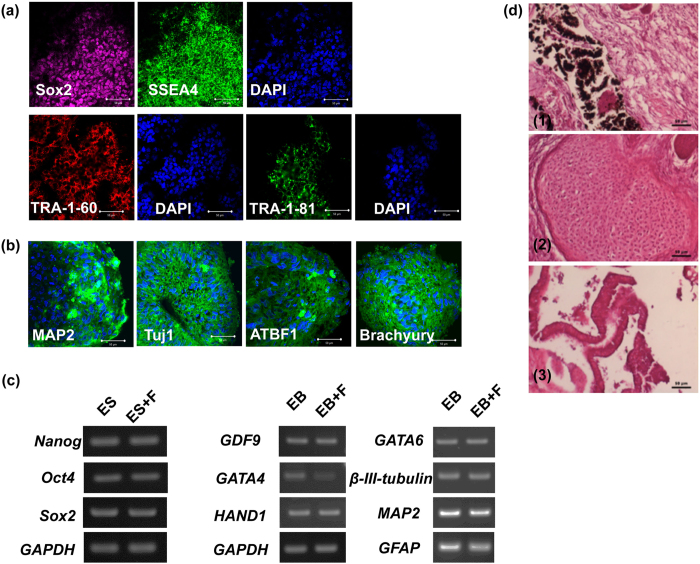
hESC/MEF following β-catenin antagonist (FH535) treatment maintained pluripotency. (**a**) Immunocytochemistry of hESC/MEF after 10 μM FH535 for 8 hours with pluripotency markers. (**b**) Immunocytochemistry of EB derived from hESC/MEF with three germ layers markers. Three germ layers markers: ectoderm (MAP2, tuj1), endoderm (ATBF1) and mesoderm (brachyury). (**c**) RT-PCR analysis of pluripotency genes (*OCT4, sox2, nanog*) and differentiation genes specific for germ cell (*GDF9*), *endoderm (GATA4*), *mesoderm (HAND1, GATA6*) and *ectoderm (β-III-tubulin, MAP2, GFAP*) were observed. *GAPDH* was adopted as a control. (**d**) Hematoxylin and eosin staining of teratoma formed by hESC/MEF treated with FH535 (1: ectoderm; 2: mesoderm; 3: endoderm). ES: human embryonic stem cell, F: FH535, EB: embryoid body. Scale bar = 50 μm. All cropped gels were run under the same experimental conditions in (**c**).

The effect of inhibition of *c*-myc by Wnt/β-catenin signaling on pluripotency of hES/MEF was investigated. Figure 5 illustrates the experimental results. Pluripotency proteins such as Sox2, SSEA4, Tra-1-60 and Tra-1-81 were expressed in the FH535-reated hES/MEF (Fig. 5a). Expressions of proteins representing three germ layers, *i.e*. MAP2 and Tuj1 for ectoderm, ATBF1 for endoderm, and Brachyury for mesoderm, were evident following a 5-day differentiation of EB (Fig. 5b). Expressions of pluripotency genes such as *OCT4, SOX2* and *NANOG* were also observed. Additionally, mRNA representing germ cells (*GDF9*), endoderm (*GATA4*), mesoderm (*HAND1, GATA6*) and ectoderm (*β-III-Tubulin, MAP2* and *GFAP*) were noted with levels equivalent to those without FH535 treatment (Fig. 5c). The rare teratoma formed by xenograft of hES/MEF with FH535 also showed three germ layers of differentiation (Fig. 5d), revealing that pluripotency was maintained.

## Discussion

Our previous study found for the first time that hESC lost their ability of teratoma formation upon co-culturing with HUCMSC, but regained the tumor forming activity after shifting back to the MEF co-culture^3^. The hESC/HUCMSC coculture indicated down-regulation of *c*-myc. This study further found that *c*-myc is down-regulated via the β-catenin signaling pathway. A TCF/LEF reporter assay and ChIP assay were performed to confirm the binding of β-catenin to the promoter of *CMYC* in hESC/MEF culture. Inhibition β-catenin with FH535 or DKK1 down-regulated both mRNA and protein expression of *CMYC*, and reduced teratoma formation by 79%. Conversely, activation of β-catenin signaling with LiCl or BIO induced an up-regulation of c-myc in the hESC/HUCMSC culture, and increased the teratoma formation from 0% to 28.6%. These findings indicate that the β-catenin/*c*-myc signaling pathway is largely responsible for the tumorigenicity of hESC.

Our previous study of hESC co-cultured with HUCMSC found downregulation of both Oct4 and *c*-myc as compared to hESC with MEF feeder^3^. Oct4 is reportedly involved in the formation of multiple cancers and their stem cells, such as colorectal^18^, liver^19^, cervical^20^,^21^, oral^22^ and ovarian cancer^23^, where its down-regulation is associated with slowed tumor progression and proliferation^24^. This may explain the incomplete recovery of tumorigenesis after adding LiCl to resume *c*-myc expression.

The GSK3 inhibitor LiCl and BIO stabilize the intracytoplasmic β-catenin protein, preventing its degradation by proteasome^25^. LiCl helps maintain the pluripotency in hESC by enhancing the β-catenin signaling^26^. Consistent with that finding, this study found that treating the HUCMSC-fed hES with LiCl could resume the expression of β-catenin and transcription of *CMYC*, and eventually resume the teratoma formation activity.

Our result is consistent with a previous report that Wnt/β-catenin signaling pathway plays a fundamental role in modulating the tumorigenicity of ESC-derived retinal progenitors^27^. Their investigation found that WNT signaling-activated TCF7-SOX2-NESTIN cascade was responsible for the tumor formation. Our study further identified *c*-myc as the major oncogenic effector of Wnt/β-catenin signaling in hESC tumorigenesis.

C-myc is a direct transactivating target of β-catenin^13^, and is one of the earliest-found oncogens. It plays a significant role in regulating cell proliferation, differentiation, stemness, senescence and tumor invasiveness^28^. In embryonic stem cells, *c*-myc is a universal amplifier of expressed genes^29^. It also plays a significant role in transcriptional regulation and matainance of the pluripotency in hESC^30^. This study found that inhibiting suppresses *CMYC* is the major mediator tumorigenic signalin of β-catenin in the hESC/MEF culture.

The teratogenicity of pluripotent embryonic stem cells has inhibited their clinical application in regenerative cellular therapy. Inhibition of β-catenin signaling could reduce teratoma formation of hESC, potentially enabling the development of safer cellular therapy than using therapy with hESC with full teratoma formation capability. Although β-catenin pathway also plays a fundamental role in stem cells self-renewal and maintenance of stem cell properties^31^, this study found that inhibition of β-catenin with FH535 does not compromise the pluripotency.

The conventional method of preventing teratoma formation in hESC cellular therapy is to apply this therapy only on the differentiated cells. Undifferentiated cells are eliminated by treating them with chemical inhibitor (YM155) to down-regulate survivin signaling^32^, with antibodies, small molecules, anti-angiogenic agents, or with suicide genes for elimination^33^. This study demonstrated for the first time that FH535 can be utilized to reduce teratogenesis in cultured hESC before induction of differentiation. Adding the beta-catenin inhibitor FH535 reduced teratoma formation by 79%. Meanwhile, researchers have investigated targeting β-catenin signaling as a novel treatment for multiple cancers such as those of the breast^34^, pancreas^35^, esophagus^36^ and liver^37^. This study recommends modulating β-catenin to reduce the risk of teratoma formation in hESC transplantation.

The non-tumorigenesis of HUCMSC coculture has many possible factors in the upstream signal of β-catenin. WIF (or sFRP) may be secreted to counteract the Wnt-Frizzled binding and down-regulate beta-catenin signaling^38^. DKK1 and SOST/WISE proteins can also bind to LRP5/6 to prevent Frizzled-LRP6 complex formation^38^. DKK1 secreted from HUCMSC was found to inhibit breast cancer cell growth^39^. This study found that DKK1 could significantly reduce nuclear β-catenin and c-myc expression of hESC/MEF. While the function of molecules in HUCMSC secretome is still largely unknown^40^,^41^,^42^, this study found that DKK1 is likely to be the major tumor suppressor secreted in the hESC/HUCMSC coculture.

In summary, this study reveals that the hESC/HUCMSC co-culture can confer a non-tumorigenesis phenotype of hESC. Down-regulation of the β-catenin/*c*-myc signaling inhibits tumor formation. Inhibition of this signaling by β-catenin inhibitor could markedly reduce the incidence of teratoma formation in the conventional hESC/MEF co-culture system.

## Methods

### Culture and Passage of hESC

The Research Ethics Committee of Buddhist Tzu Chi General Hospital (IRB 100–166) approved the protocols for collecting and using human umbilical cord. Written informed consent was obtained from the pregnant women before labor. The methods were performed in accordance with the relevant guidelines, including any relevant details. The TW1 hESC was obtained from the Food Industry Research and Development Institute of Taiwan, and maintained on the mitomycin-C treated MEF (hESC/MEF) or HUCMSC (hESC/HUCMSC) following a previously reported protocol^3^. The experiment was performed using knock-out (KO) Dulbecco’s modified Eagle’s medium (DMEM) and 20% (v/v) KO Serum Replacement containing 2 mM glutamine, 10 nM non-essential amino acids (all from Invitrogen, Thermo Fisher Scientific, Waltham, MA, USA, https://www.thermofisher.com/), 50 μM B-mercaptoethanol (Sigma-Aldrich, St. Louis, MO, http://www.sigmaaldrich.com) and 4 ng/ml basic fibroblast growth factor (bFGF). The medium was changed daily, and the hESC was passed each week.

To prepare the hESCs with different feeders, hESC was first co-cultured with MEF (hESC/MEF), and the established hESC clusters were transferred to the HUCMSC feeder as the hESC/HUCMSC co-culture. For the reverse co-culture, hESC was further transferred to the MEF feeder as the hESC/MHM. All the feeder transfers were performed after five passages in the previous feeder. The hESC/MHM were also maintained for more than five passages before investigation.

### Chemicals

FH535, LiCl and BIO (6-bromoindirubin-3′-oxime) were purchased from Sigma-Aldrich. DKK1 was purchased from R & D systems.

### Quantitative RT-PCR and RT-PCR

RNA for all qRT-PCR and RT-PCR analysis was prepared using Trizol (Invitrogen) and quantified. 500ng of RNA was DNAase-treated using DNaseI amplification grade (Invitrogen). The first strand of cDNA was synthesized by a SuperScript III One-Step RT-PCR kit (Invitrogen) following the manufacturer’s instructions. All PCR samples were analyzed by electrophoresis on 2% agarose gel containing 0.5 μg/ml ethidium borome (Sigma). The quantitative RT-PCR (qRT-PCR) analysis adopted FastStart universal SYBR green master (ROX, Roche, Basel, Switzerland, https://lifescience.roche.com) gene expression assays in an ABI Step One Plus system (Applied Biosystems, Thermo Fisher Scientific), with *GAPDH* as an internal control. Table 3 shows the sequences of primers and product size.

### Chromatin immunopreciptation (ChIP) assay

SimpleChIP Enzymatic Chromatin IP kit (Cell Signaling) was adopted for ChIP assay for beta-catenin-CMYC promoter binding. The assay with beta-catenin antibody was performed with 4 × 10^6^ hES cells cultured with different feeders, according to the manufacturer’s instructions. The bound *CMYC* sequences were quantified by qPCR after preparing ChIP DNA. The primer sequences of *CMYC* promoter are listed as below: forward GTG AAT ACA CGT TTG CGG GTT AC; reverse AGA GAC CCT TGT GAA AAA AAC CG.

### Western blot assay

The cells were lysed in the lysis buffer (150 mM NaCl, 50 mM Tris–HCl, pH 7.4, 1% Nonidet P-40) plus proteinase inhibitor cocktail (Roche). The proteins were electrophoresed on 10% sodium dodecyl sulfate-polyacrylamide gel electrophoresis, then transferred to a nitrocellulose membrane (Hybond-C Super; GE healthcare, Little Chalfont, UK, http://www.gehealthcare.com/). The membranes were incubated with specific monoclonal antibodies. The secondary antibody was HRP-conjugated goat anti-mouse IgG (Jackson Immuno-Research Laboratories, West Grove, PA, https://www.jacksonimmuno.com/). The bound antibodies were detected by enhanced chemiluminescence reagents (ECL; GE).

### Extraction of cytoplasmic/nuclear proteins from hESCs

ReadyPrep protein extraction kit (cytoplasmic/nuclear, Bio-Rad) was adopted to isolate protein from the nucleus and cytoplasm. The isolation procedures were conducted according to the manufacturer’s instructions. The resulted cytoplasmic/nuclear protein were analysed by Western blot.

### TCF/LEF report assay

The TCF/LEF signal reporter assay kit (Qiagen) was adopted to demonstrate the transactivating activity of β-catenin. Briefly, one day before transfection, ES cells were seeded at a density of 30,000 cells per well into a 96-well plate in 100 μl of medium. Next day, 1 μl of TCF/LEF luciferase reporter (component A) was transfected from each well into cells. After 24 h of transfection, 55 μl of Luciferase reagent per well were added and shaken at room temperature for 15 min, and firefly luminescence was measured by a luminometer. 55 μl of Stop & Glo reagent per well were added and rocking at room temperature for 15 min, and Renilla luminescence was measured. To obtain the normalized luciferase activity for TCF/LEF reporter, the background luminescence was calculated as the ratio of firefly luminenscence from the TCF/LEF reporter to Renilla luminescence, and subtracted from the control Renilla luciferase vector. Graphs were plotted from data obtained as a mean of three independent experiments, with standard deviation shown as error bars.

### Xenograft in immune-compromised mice

All animal works were in accordance with protocols approved by the Institutional Animal Care and Use Committee at the Buddhist Tzu Chi General Hospital. All methods were performed in accordance with the relevant guidelines and regulations. For the tumorigenesis assay, hESC were removed from the feeder with mechanical slicing using glass capillaries, then pelleted and resuspended in PBS. For the xenograft, 5 × 10^5^ cells mixed with Matrigel (1:1) were injected into the back subcutaneous tissue of 6–8-week-old female non-obese diabetic-severe combined immunodeficiency (NOD-SCID) mice. Tumor formation was followed up by palpation. The resulting tumors were dissected, fixed, embedded in paraffin and processed for histological examination.

### Immunohistochemistry

The hESC colonies were cultured on chamber slides (Nunc, Thermo Fisher Scientific) in culture dishes with feeder cells, then subjected to immunohistochemistry 3–7 days following passage. Cells were fixed with 4% paraformaldehyde, and permeabilized with 0.1% Triton X-100, blocking with 4% normal goat serum, then treatment with primary antibodies such as Sox2, stage-specific embryonic antigen-4 (SSEA4), TRA-1-60 and TRA-1-81 (ES Cell Characterization Kit; Chemicon, EMD Millipore, Billerica, MA, www.emdmillipore.com/).

For differentiation of hESC, embryoid body (EB) formation was performed for 5 days. The resulting EB was plated on gelatin-treated chamber slides and fixation. Antibodies specific for three germ layers, namely ectoderm [microtubule associated protein 2 (MAP2), tuj-1], mesoderm (brachyury) and endoderm [AT motifi-binding factor 1 (ATBF-1)], were identified.

### Statistical analysis

All analyses were conducted by the software IBM SPSS Statistics version 21 (IBM, Armonk, NY, USA, http://www.ibm.com/). Data are shown as mean ± SE. The means were compared by one-way ANOVA followed by Tukey correction. The level of statistical significance was set at *p* < 0.05.

## Additional Information

**How to cite this article**: Chang, Y.-H. *et al*. WNT/β-Catenin signaling pathway regulates non-tumorigenesis of human embryonic stem cells co-cultured with human umbilical cord mesenchymal stem cells. *Sci. Rep.* **7**, 41913; doi: 10.1038/srep41913 (2017).

**Publisher's note:** Springer Nature remains neutral with regard to jurisdictional claims in published maps and institutional affiliations.

## Figures and Tables

**Table 1:** Xenograft generation of hESC cultured on HUCMSC with or without treated with LiCl.

	Tumor		No tumor		Total	P-value of chi-square
	N	%	N	%		
ES/HUCMSC	0	0	21	100	21	0.056
ES/HUCMSC + LiCl	2	28.6	5	71.4	7	

ES: human embryonic stem cell.

HUCMSC: human umbilical cord mesenchymal stem cells.

**Table 2:** Xenograft generation of hESC cultured on MEF with or without treated with FH535.

	Tumor		No tumor		Total	P-value of chi-square
	N	%	N	%		
ES/MEF	4	100	0	0	4	0.011
ES/MEF + FH535	3	21.4	11	78.6	14	

ES: human embryonic stem cell.

MEF: mouse embryonic fibroblast.

**Table 3:** Primer set of various genes adopted in quantitative and RT-PCR.

Gene	Sense (5′-3′)	Antisense (5′-3′)	Product size (bp)
*GAPDH*	GGCAGCAGCAAGCATTCCT	GCCCAACACCCCCAGTCA	226
*c-myc*	AAACACAAACTTGAACAGCTAC	ATTTGAGGCAGTTTACATTATGG	188
*sox2*	CCC CCG GCG GCA ATA GCA	TCG GCG CCG GGG AGA TAC AT	448
*oct4*	CTT GCT GCA GAA GTG GGT GGA GGA	CTG CAG TGT GGG TTT CGG GCA	169
*nanog*	AGT CCC AAA GGC AAA CAA CCC ACT TC	TGC TGG AGG CTG AGG TAT TTC TGT CTC	161
*GATA4*	TCC CTC TTC CCT CCT CAA AT	TCA GCG TGT AAA GGC ATC TG	194
*HAND1*	TGC CTG AGA AAG AGA ACC AG	ATG GCA GGA TGA ACA AAC AC	274
*GATA6*	CCT CAC TCC ACT CGT GTC TGC	GTC CTG GCT TCT GGA AGT GG	225
*βIII tubulin*	CAG AGC AAG AAC AGC AGC TAC TT	GTG AAC TCC ATC TCG TCC ATG CCC TC	227
*MAP2*	GCA TGA GCT CTT GGC AGG	CCA ATT GAA CCC ATG TAA AGC C	194
*GFAP*	AGG GCT GAC ACG TCC AC	GCC TTA GAG GGG AGA GGA G	132
*GDF9*	TAG TCA GCT GAA GTG GGA CA	ACG ACA GGT GCA CTT TGT AG	277
*esrrb*	CAAGAAGCTCAAGGTGGAGAAGGAGGAG	CGGTCTGTCCGTTTGTCTGTCTGTAGGT	200
*Lin28*	TGCACCAGAGTAAGCTGCAC	CTCCTTTTGATCTGCGCTTC	189
*Klf4*	CTCCTTTTGATCTGCGCTTC	ATGTGTAAGGCGAGGTGGTC	169
